# Comparing the efficacy of syngeneic iliac and femoral allografts with iliac crest autograft in a rat model of lumbar spinal fusion

**DOI:** 10.1186/s13018-020-01936-8

**Published:** 2020-09-15

**Authors:** Christina Holmes, Benjamin D. Elder, Wataru Ishida, Alexander Perdomo-Pantoja, John Locke, Ethan Cottrill, Sheng-Fu L. Lo, Timothy F. Witham

**Affiliations:** 1 Department of Chemical and Biomedical Engineering, Florida A&M University-Florida State University College of Engineering, Tallahassee, FL USA; 2 Department of Neurosurgery, The Johns Hopkins University, School of Medicine, Baltimore, MD USA; 3 Department of Neurosurgery, Mayo Clinic, Rochester, MN USA

**Keywords:** Spinal fusion, Animal model, Autograft, Allograft, Bone marrow cells

## Abstract

**Background:**

Despite widespread use of femoral-sourced allografts in clinical spinal fusion procedures and the increasing interest in using femoral reamer–irrigator–aspirator (RIA) autograft in clinical bone grafting, few studies have examined the efficacy of femoral grafts compared to iliac crest grafts in spinal fusion. The objective of this study was to directly compare the use of autologous iliac crest with syngeneic femoral and iliac allograft bone in the rat model of lumbar spinal fusion.

**Methods:**

Single-level bilateral posterolateral intertransverse process lumbar spinal fusion surgery was performed on Lewis rats divided into three experimental groups: iliac crest autograft, syngeneic iliac crest allograft, and syngeneic femoral allograft bone. Eight weeks postoperatively, fusion was evaluated via microCT analysis, manual palpation, and histology. In vitro analysis of the colony-forming and osteogenic capacity of bone marrow cells derived from rat femurs and hips was also performed to determine whether there was a correlation with the fusion efficacy of these graft sources.

**Results:**

Although no differences were observed between groups in CT fusion mass volumes, iliac allografts displayed an increased number of radiographically fused fusion masses and a higher rate of bilateral fusion via manual palpation. Histologically, hip-derived grafts showed better integration with host bone than femur derived ones, likely associated with the higher concentration of osteogenic progenitor cells observed in hip-derived bone marrow.

**Conclusions:**

This study demonstrates the feasibility of using syngeneic allograft bone in place of autograft bone within inbred rat fusion models and highlights the need for further study of femoral-derived grafts in fusion.

## Background

Spinal fusion is an increasingly common procedure used to treat diverse pathologies arising from spinal trauma, degenerative diseases, deformity, infection, and tumors. Although generally successful in most patients, fusion fails in up to 35% of cases, resulting in significant patient morbidity, the need for additional procedures, and increased health care costs [1]. Over the past decade, a wide range of treatment options have been explored to prevent fusion failure, or pseudarthrosis, with much of this research employing pre-clinical animal models.

The rat posterolateral spinal fusion model has become an increasingly popular experimental model to assess the efficacy of novel fusion treatments [2]. Compared to other commonly used animal models in spinal fusion research, such as rabbits, sheep, goats, pigs, dogs, and monkeys, the rat model presents a number of key advantages [3]. Rats are lower cost, facilitate shorter operation times and higher-throughput studies, and most importantly, enable more in-depth analyses of the biology underlying fusion due to a wider availability of cellular and molecular tools. Various fusion therapies have been studied in rat models, including a range of bone graft substitute and extension materials, systemic and localized delivery of osteogenic growth factors and/or osteoporosis therapies, and stem cell transplantation therapies [4–14].

As autograft iliac crest bone remains the clinical “gold standard” in fusion procedures, many rat fusion studies employ this graft source as a control group. However, autograft iliac crest bone is limited in supply, can be difficult to harvest at a consistent volume, and its harvest leads to both additional surgical time per animal and donor site morbidity. These issues can be circumvented by employing inbred rat strains, which allow for the use of syngeneic allografts in place of autograft bone. The ability to employ syngeneic allografts also enables evaluation of the fusion efficacy of bone grafts derived from different skeletal sites. Remarkably, despite the widespread use of femoral-sourced allografts in clinical spinal fusion procedures and the increasing interest in using femoral reamer–irrigator–aspirator (RIA) autograft in clinical bone grafting procedures, the fusion efficacy of femoral- and iliac crest-derived grafts has yet to be compared in the spine [15–18]. In this study, we thus directly compared the use of autograft iliac crest bone to syngeneic femoral and iliac crest allograft bone in the rat model of posterolateral lumbar spinal fusion. Since a key element to the clinical success of autograft bone is the presence of osteogenic cells, we also performed in vitro analysis of the colony-forming and osteogenic capacity of bone marrow cells derived from rat femurs and hips to determine whether this correlated with the fusion efficacy of these graft sources.

## Methods

### Animals

The following study was approved by the Institutional Animal Care and Use Committee at Johns Hopkins University School of Medicine (RA14M347). Experimental animals were housed in a specific pathogen-free facility and fed a standard diet. All animals used in this study were 6–9-week old female Lewis rats (100–180 g).

### Surgical procedures

Single-level bilateral posterolateral intertransverse process lumbar spinal fusion surgery was performed, as described previously [19, 20], on 53 host rats divided into three experimental groups: [A] iliac crest autograft (*n* = 16), [B] syngeneic iliac crest allograft (*n* = 19), and [C] syngeneic femoral allograft bone (*n* = 18). Briefly, host rats were anesthetized via intraperitoneal (IP) injection of ketamine (36 mg/kg) and xylazine (4 mg/kg). The surgical site was shaved and prepped with 70% ethanol and povidone-iodine, and sterile gloves and masks were used by all surgical personnel. The surgical procedure was performed using an operating microscope or surgical loupes at × 2.5 to × 10 magnification. The L4 to L5 vertebral levels were identified by palpation and anatomical landmarks. A dorsal midline skin incision was made centered over the L4-L5 spinous process, and a self-retaining retractor was utilized to retract skin edges. Two paramedian fascial incisions were then made through the lumbar fascia. The intermuscular plane was established between the multifidus and longissimus muscles to expose the transverse processes of L4 to L5 as well as the inter-transverse membrane. Decortication of the transverse processes and lateral pars/facet joints was performed with a motorized burr. The appropriate graft was placed over the entire fusion bed space (L4 to L5) on each side of the spine.

In the autograft group [A], the midline incision was extended to the sacrum, and dissection was taken to the iliac crest on each side, and following subperiosteal dissection over the posterior aspect of the iliac crest, a small volume of corticocancellous autograft was harvested with a rongeur. In the syngeneic allograft groups, hips (the ilium down to the acetabulum) [B] and femurs [C] were sterilely isolated from freshly euthanized littermate donors and placed on ice, immediately prior to fusion surgery. In most cases, harvested bone grafts were weighed on a sterile scale. In all cases, isolated bones were morselized with a rongeur prior to implantation to create a homogenous distribution of corticocancellous graft material over the fusion bed.

Fascia and skin were closed in layers with 5-0 absorbable sutures (Polysorb, Medtronic, Minneapolis, MN, USA). Normal saline was administered intraperitoneally after wound closure as needed. Rats were maintained on a heating pad until spontaneous ambulation was observed. Buprenorphine (0.01 mg/kg) was administered subcutaneously every 24 h for 2 days. After surgery, rats were closely monitored for any sign of nerve palsy, hemiparesis, or infection as well as any changes in general condition. All animals were euthanized 8 weeks post-surgery and spines were harvested.

### Radiographical analysis

Harvested lumbar spines were imaged using a nanoSPECT/CT Small Animal Imager (Mediso Medical Imaging Systems, Budapest, Hungary). The coronal CT images were evaluated by two authors in a blinded fashion. Each fusion mass (i.e., two per animal, one on each side) was graded as either fused (i.e., robust fusion between L4-5 was observed), partially fused (i.e., some narrowing of the fusion mass between L4-5 was present), or non-fused (i.e., there was a discontinuity of fusion mass between L4-5). Axial cross sections were also generated to quantitatively calculate fusion mass volume via ImageJ software (US National Institutes of Health, Bethesda, MD) and the Volumest plugin as previously described [20–22].

### Manual palpation

Soft tissue surrounding the L3-L6 region of harvested spines was gently removed. Two blinded independent observers manually palpated the fusion site (L4–L5) to evaluate biomechanical fusion. Palpation was scored as either bilaterally fused (i.e., no segmental motion compared to the adjacent levels) or non-fused (i.e., similar segmental motion across the index level compared to the adjacent levels).

### Histology

Harvested spine samples were fixed in 4% paraformaldehyde overnight, decalcified in Rapid Bone Decalcifier (American MasterTech Scientific, Lodi, California) for 8 h, dehydrated by ethanol series (70%, 95%, 100%), and embedded in paraffin. Serial coronal sections (10-μm thick) across the level of the fusion masses were cut, deparaffinized in xylene, and subsequently rehydrated in a descending ethanol series (100%, 95%, and 70%). Hematoxylin and eosin (H&E) staining and Masson’s trichrome staining were conducted to evaluate the formation of bone, cartilage, and osteoid.

### Bone marrow cell isolation and culture

Nine donor rats were euthanized, and femurs and ilia were isolated, dissected, and cleaned in a sterile biological safety cabinet. Bones were cut into smaller pieces, crushed using a sterile mortar and pestle, and washed several times in chilled culture medium (Dulbecco’s Modified Eagle Medium (DMEM; high glucose; Gibco, USA) supplemented with 10% fetal calf serum (FCS) and 1% (v:v) penicillin–streptomycin) to isolate bone marrow cells. The resulting cell suspension was passed through a 100-μm nylon mesh filter and subsequently underwent hemolysis and centrifugation. Recovered nucleated cells were enumerated, re-suspended, and either used in limiting dilution assays or cultured at 37 °C in humidified air with 5% CO_2_. The first media exchange was performed ∼ 72 h after plating, with subsequent media exchanges every 2–3 days.

### Limiting dilution colony-forming unit fibroblast (CFU-F) assay

The frequency of mesenchymal progenitor cells within hip-derived and femur-derived bone marrow was determined via limiting dilution colony-forming unit fibroblast (CFU-F) assays. Briefly, cultures were initiated with freshly isolated bone marrow cells at densities of 3 × 10^5^, 1 × 10^5^, 3 × 10^4^, 2.5 × 10^6^, and 5 × 10^6^ cells/well in 96/well plates in culture media, with 10 replicate wells per density per rat (*n* = 7 for femur, *n* = 6 for hip). On day 10 of culture, plates were fixed with methanol and stained with Crystal Violet. The total number of wells containing at least 1 fibroblastic CFU-F colony of 10 or more cells (representing at least three population doublings) at each density was enumerated using microscopic observation. CFU-F frequencies were calculated using the online ELDA tool available at http://bioinf.wehi.edu.au/software/elda/ [23].

### Osteogenic differentiation assay

To assess the osteogenic differentiation capacity of hip-derived and femur-derived bone marrow, passage 1 (P1) cells from 8 donor rats were seeded in triplicate at a density of 50,000 cells/cm^2^ in either osteogenic media (DMEM (low glucose; Gibco, USA) supplemented with 10% FCS, 1% penicillin–streptomycin, 10 mM β-glycerophosphate (Sigma), and 50 μM l-ascorbic acid-2-phosphate) or in standard culture media (as a negative control) and cultured for 21 days. Mineralization was assessed via alizarin red S staining. Briefly, samples were washed twice with PBS, fixed with 3.7% formaldehyde for 20 min, washed again three times, subsequently incubated for 10 min with 40 mM alizarin red S (Sigma), and then washed extensively before imaging. Subsequently, alizarin red S was eluted from stained cultures for quantification via incubation with 10% acetic acid at room temperature with shaking for 30 min, followed by cell scraping and transfer to Eppendorf microcentrifuge tubes. The resulting samples were vortexed for 30 s, heated at 85 °C for 10 min, incubated on ice for 5 min, and subsequently centrifuged at 20,000×*g* for 15 min. Two hundred microliters of the resulting supernatant was transferred to a new microcentrifuge tube and neutralized with 75 μl of 10% ammonium hydroxide. One hundred fifty microliters was transferred to a 96-well plate, and absorbance was measured at 405 nm using a microplate reader (PerkinElmer VICTOR3).

### Statistical analysis

In the case of three experimental groups (e.g., comparison of graft weights, CT volumes), intergroup comparisons of continuous variables were performed via one-way ANOVA (parametric) or Kruskal-Wallis tests (non-parametric), while categorical data sets were tested via the *χ*^2^ test. In the case of two experimental groups (e.g., comparison of cell yield, alizarin red staining), intergroup comparison was performed via paired Student’s *t* test. All reported *p* values are 2-sided, and *p* values < 0.05 were considered to be statistically significant. All statistical analyses were performed using GraphPad Prism 6.0 (La Jolla, CA, USA) with the exception of analysis of CFU-F frequency, which was performed using the online ELDA tool available at http://bioinf.wehi.edu.au/software/elda/ [23].

## Results

### Fusion assessment

**Fig. 1:**
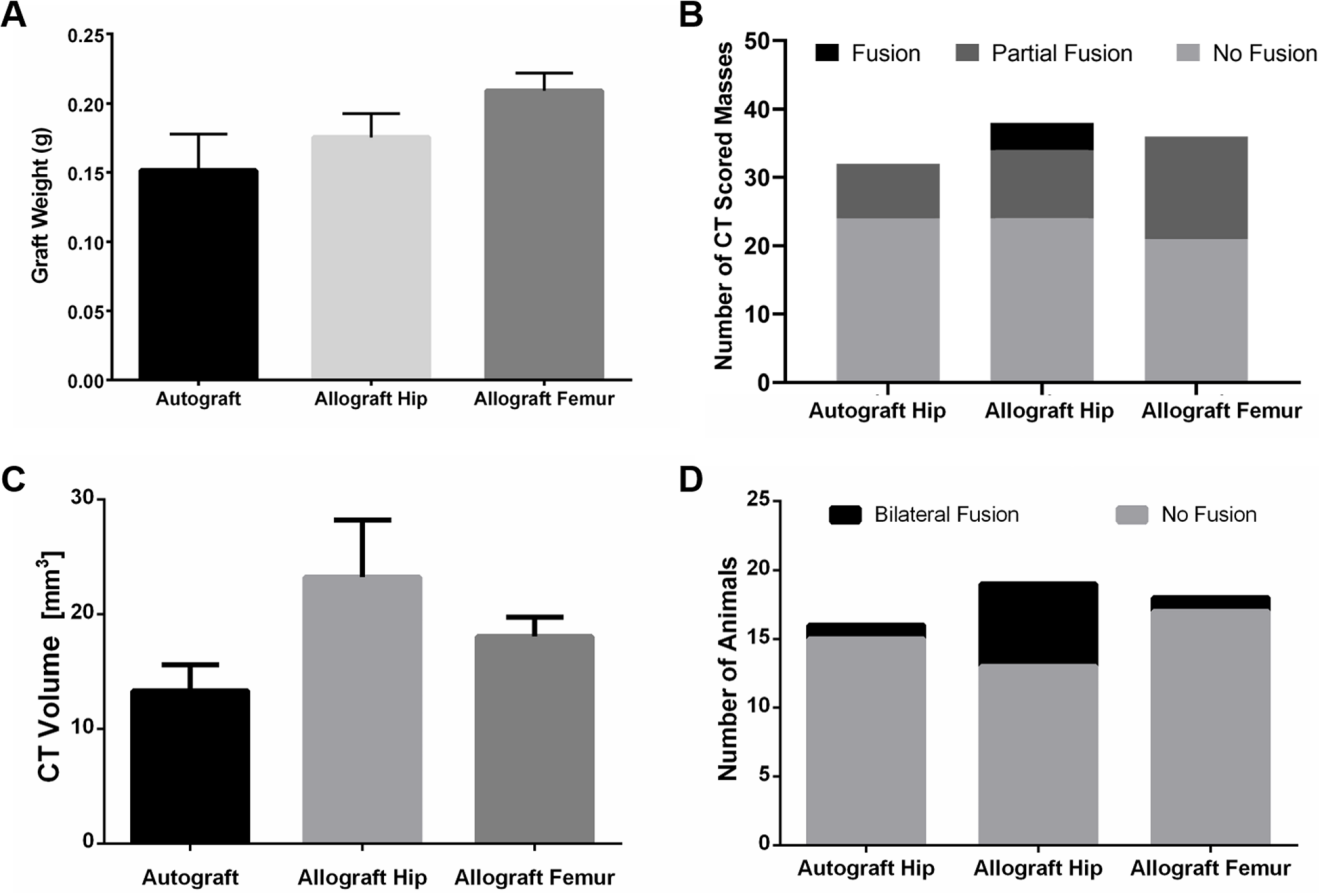
Fusion assessment. Comparisons of **a** pre-implantation graft weight, **b** number of CT fusion scored masses, **c** CT volumes of fusion masses, and **d** number of fused animals via manual palpation. CT fusion was scored as either fused if robust fusion between L4-5 was observed, partially fused if some narrowing of fusion mass between L4-5 was present, and non-fused if there was a significant discontinuity of the fusion mass. CT volumes of fusion masses were calculated using the Volumest plug-in for Image J. Animals were scored via manual palpation as bilaterally fused (little to no motion across the operated joint) or non-fused (no reduced motion across the operated joint compared to the next upper level). Data presented as mean ± SEM. For graft weight: iliac crest autograft (*n* = 12), syngeneic allograft hip (*n* = 14), and syngeneic allograft femur (*n* = 14). For all other assessments: iliac crest autograft (*n* = 16), syngeneic allograft hip (*n* = 19), and syngeneic allograft femur (*n* = 18)

Rats recovered well from surgery and did not exhibit any significant complications. There was no significant difference among the mean pre-implantation graft weights which were 0.151 ± 0.092 g, 0.175 ± 0.064 g, and 0.209 ± 0.048 g for grafts derived from autograft hip, allograft hip, and allograft femur, respectively (*p* = 0.113, Fig. 1a).

**Fig. 2:**
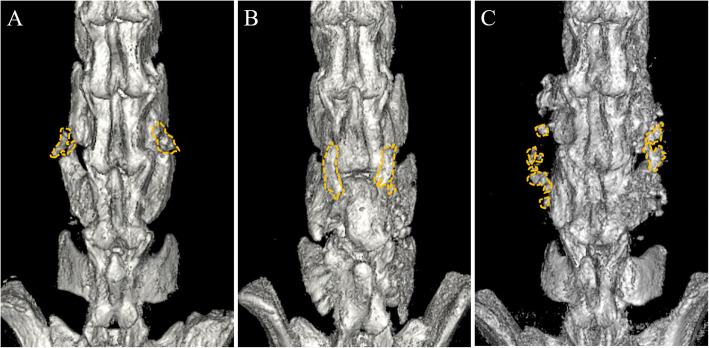
Representative renderings of μCT images. Autograft hip (**a**, left), allograft hip (**b**, center), and allograft femur (**c**, right) grafting groups. Bone formation in the intertransverse space was seen in each experimental group (yellow dashes surrounding only the newly formed bone located in the area between transverse processes). Although allograft hip (**b**) yielded higher fusion mass volumes, this difference was not statistically significant. Hip-derived fusions tended to appear as single intertransverse masses, while femoral allografts exhibited bone formation with a scattered pattern

Analysis of microCT images yielded average fusion mass volumes of 13.3 ± 8.9 mm^3^ in the autograft group, 23.2 ± 21.6 mm^3^ in the iliac allograft group, and 18.0 ± 7.2 mm^3^ in the femoral allograft group (Figs. 1c, and 2). There were no statistically significant differences among these groups (*p* = 0.140). However, radiographic scoring of fusion masses (Figs. 1b, and 2) revealed that, while none of the fusion masses in the autograft group or allograft femur group exhibited full radiographic fusion, 10.5% of fusion masses in the iliac autograft group fully fused (*p* = 0.043). Partial radiographic fusion was observed in 25.0%, 26.3%, and 41.7% of fusion masses in the autograft, iliac allograft, and femoral allograft groups, respectively (*p* = 0.043). Similarly, manual palpation analysis of fusion masses indicated that iliac allografts yielded the highest bilateral fusion rates with 31.6% bilaterally fused, compared to 6.3% in the autograft group, and 5.6% in the femoral allograft groups (Fig. 1d) (*p* = 0.043).

**Fig. 3:**
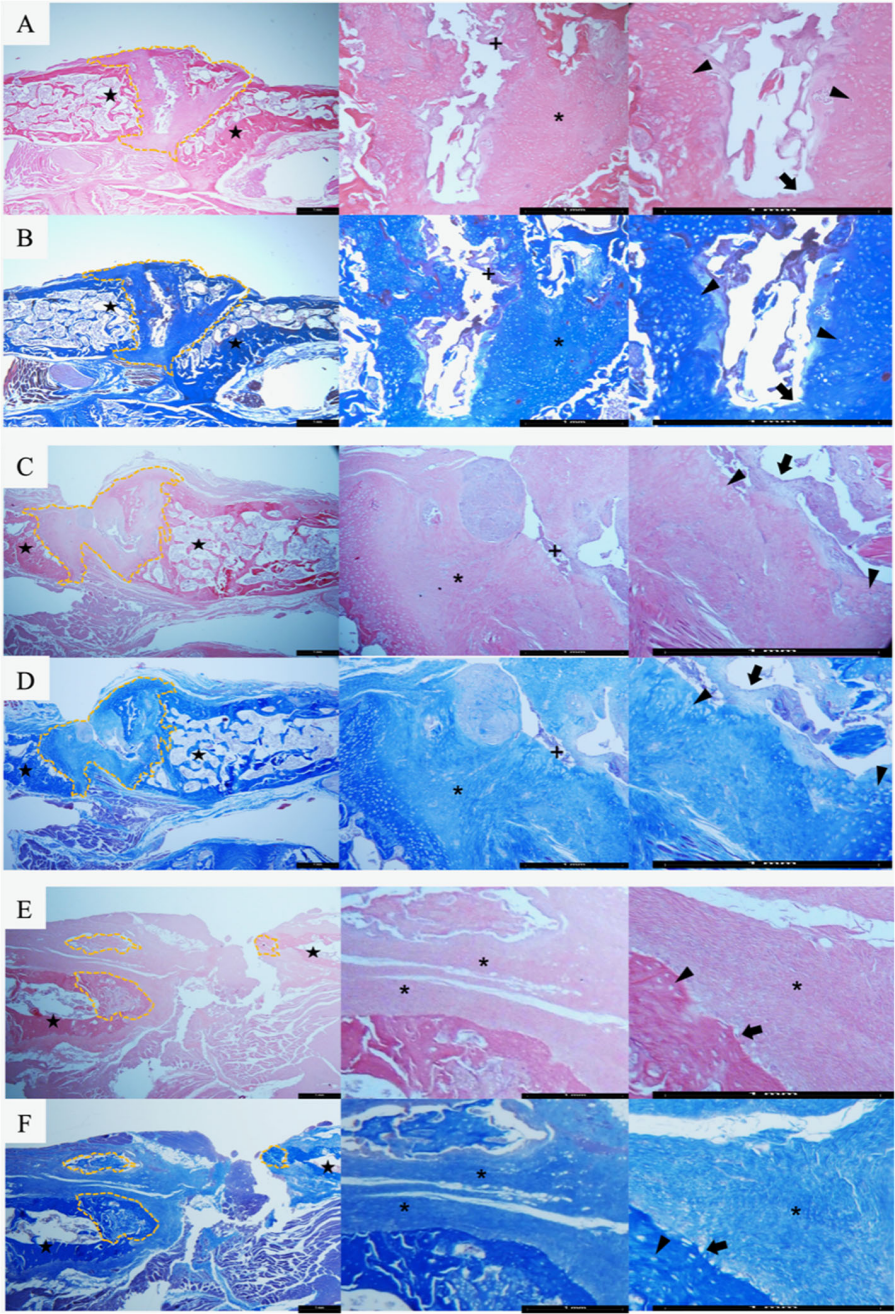
Representative histological images of fusion masses. Hematoxylin-eosin (**a**, **c**, **e**) and Masson’s trichrome (**b**, **d**, **f**) staining (× 2.5, × 10, and × 20 magnification, respectively) of representative fusion areas (yellow dashed lines) from autograft hip (**a**, **b**), allograft hip (**c**, **d**), and allograft femur (**e**, **f**) groups. Unlike the allograft femur group (**e**, **f**), fusion masses arising from hip-derived grafts (**a**, **b**, **c**, **d**) exhibited bone formation on both adjacent transverse processes (stars). Within the intertransverse space in hip-derived graft groups (**a**, **b**, **c**, **d**), bridging osteoid (*) with a high number of osteoblasts on the surface (black arrows) and abundant osteocytes within lacunae (black arrowheads), as well as incipient bone marrow (+) in the center of the fusion masses were observed. Black bar in all images represents 1 mm

Histology revealed that the iliac auto- and allograft groups displayed zones of osteointegration between grafts and both transverse processes more often than the femoral allograft group (Fig. 3). Increased osteoid deposition, number of osteoblasts, and osteocytes were observed within the fusion masses of the hip-derived graft groups, while the femur-derived graft group exhibited significant osteoid deposition but lower cellularity. Fusion masses from the hip-derived graft groups also showed more extensive areas of bone marrow than those from the femoral allograft group.

### Cellular analysis

**Fig. 4:**
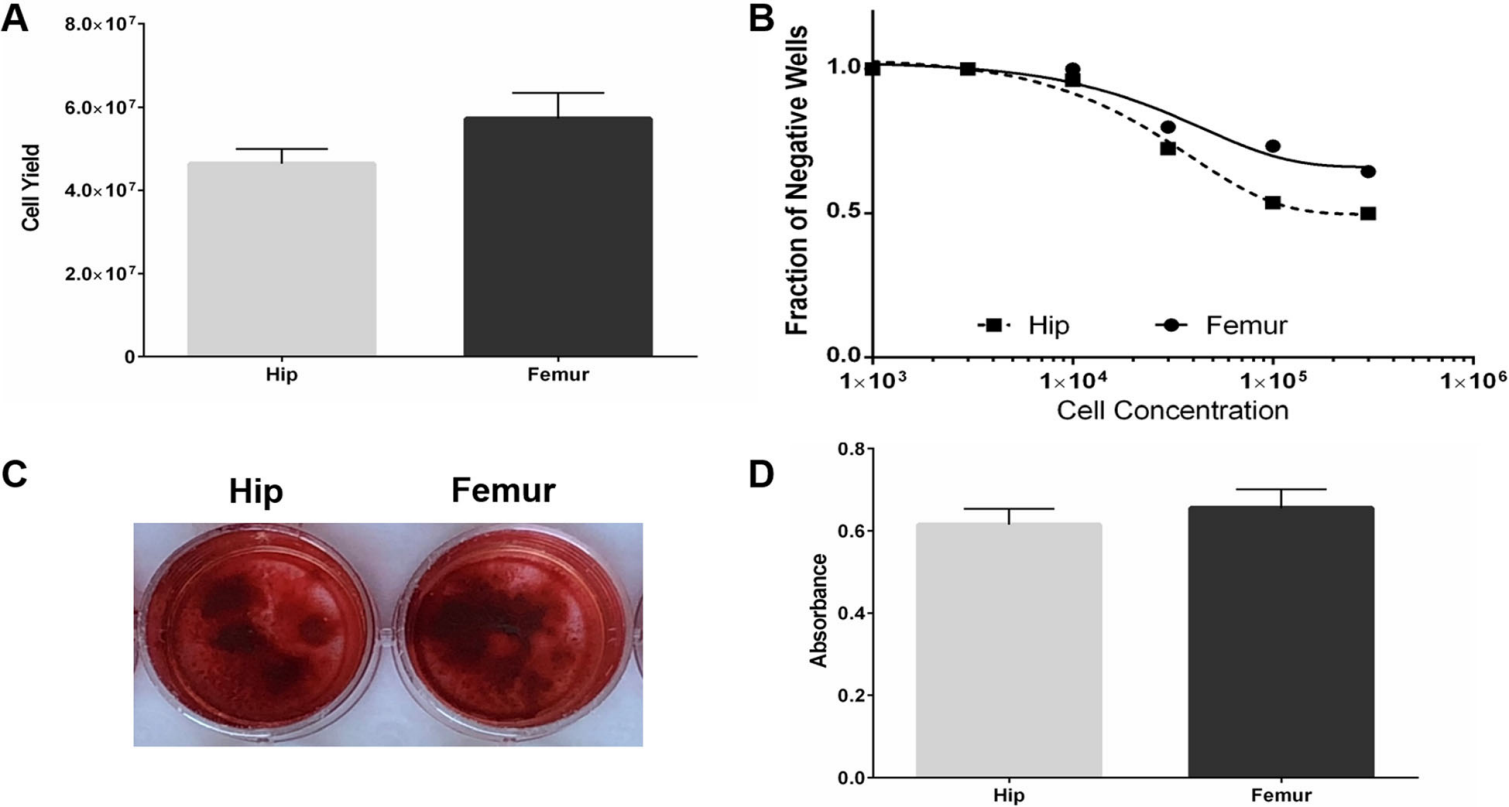
Cellular analyses. **a** Total nucleated cell yield from hips (gray bar) and femurs (black bar) (*n* = 9). **b** Limiting dilution analysis of osteogenic progenitor cells within bone marrow isolated from hips (squares) and femurs (circles). The fraction of wells without colony-forming unit fibroblast (CFU-F) colonies was plotted against the number of cells inoculated. Colonies were evaluated on day 10 (*n* = 7 for femur, *n* = 6 for hip). **c** Mineralization of hip-derived (left) and femur-derived (right) cells was qualitatively assessed via alizarin red (AR) staining, and representative images are presented. Passage 1 cells were cultured in osteogenic media for 28 days. **d** Quantification of AR staining intensity (after solubilization) from hip-derived (gray bar) and femur-derived (black bar) osteogenic cultures (*n* = 8). Data presented as mean ± SEM

Whole femurs yielded more nucleated cells than ilia (5.73 ± 1.86 × 10^7^ vs. 4.64 ± 1.07 × 10^7^ cells, *p* = 0.030; Fig. 4a). However, limiting dilution CFU-F analysis demonstrated a higher frequency of mesenchymal progenitors in hip-derived than femur-derived bone marrow cell populations (1/85,292 cells vs. 1/258,838 cells, *p* = 0.015; Fig. 4b). Hip- and femur-derived bone marrow mesenchymal progenitors displayed similar osteogenic differentiation capacity, as alizarin red S staining indicated no significant differences (*p* = 0.469; Fig. 4c, d).

## Discussion

This is one of the first studies to directly compare the efficacy of femoral vs. iliac allograft, as well as autograft vs. allograft bone, in a rat spinal fusion model. Although no statistically significant differences were observed between grafting groups in terms of initial graft weight or fusion mass volume, iliac allografts were found to yield both significantly more fully fused fusion masses via CT scoring and a significantly higher rate of bilateral fusion via manual palpation. The higher CT and palpation fusion rates observed in the iliac allograft group compared to the corresponding iliac autograft group were potentially related to the higher fusion mass volume (23.2 ± 21.6 mm^3^ vs. 13.3 ± 8.9 mm^3^) shown in the allograft group, even though this increase was not statistically significant due to high variability. Another probable contributing factor is the slight difference in overall graft compositions, as the syngeneic hip allografts were derived from the whole ilium (including elements of the acetabulum), while the autografts consisted of the iliac crest alone, thus leading to possible differences in cortical-to-trabecular ratios as well as total cellular composition and concentration. Interestingly, the greater CT and palpation scores seen in the allograft hip group compared to the femoral group were reflected in the histological observations of better integration with host bone and increased osteoblasts and osteocytes in hip-derived fusion masses in contrast to those derived from femoral grafts.

The histological differences in the study were echoed in our in vitro analyses. Hip-derived bone marrow cells exhibited a higher CFU-F frequency than femur-derived populations, thus indicating a higher concentration of the osteogenic progenitor cells than can differentiate into osteoblasts and osteocytes. This increase in hip-derived mesenchymal progenitor cell frequency also likely contributed to the improved integration with host bone observed in hip graft-derived fusion masses, as mesenchymal progenitor cells are known to recruit host cell migration via paracrine mechanisms and improve bone repair [24]. The bone-forming capacity of the hip- and femur-derived bone marrow progenitor cells, however, was found to be similar. There were no differences in osteogenic differentiation in vitro between P1 bone marrow cells derived from either bone marrow population, and histology indicated that both hip- and femur-derived graft groups yielded osteoid deposition within fusion masses in vivo.

The increased CT fusion scores observed in this study for syngeneic iliac allograft compared to autograft bone is in contrast to several clinical fusion studies which also used radiographic or CT measures of fusion to compare autograft to frozen allograft [25, 26]. For example, recent meta-analyses by several groups have found comparable lumbar fusion rates between patients treated with allograft and autograft iliac crest bone, with no significant differences in disability or pain scores [27, 28]. While this discrepancy between our observations in a preclinical animal model and clinical studies may be due to differences in species, the fresh and syngeneic nature of the allografts used in this study may also be contributing factors.

Although autologous iliac crest bone remains the “gold standard,” femoral-sourced grafts have been commonly used in spinal fusion and bone grafting procedures. In clinical fusion procedures, femoral ring and femoral dowel allografts have been widely employed as osteoconductive structural grafts in interbody procedures [29–33], while femoral head allograft has been successfully used in interbody [16, 34–36], and posterolateral lumbar procedures [15, 17]. With increasing interest in the use of femoral reamer–irrigator–aspirator (RIA) bone autograft, a number of clinical studies have recently compared the efficacy of RIA and iliac crest autograft in nonunion, posttraumatic segmental bone defect, or ankle fusion patients and have observed comparable [37–40], or increased union rates and times to union [41]. Femoral RIA autograft has also been successfully used in clinical interbody and posterolateral fusion procedures [42, 43]. Our study, which is the first to directly compare femur- and hip-derived bone grafts in spinal fusion, suggests that femur-derived grafts at least perform comparably to autograft hip-derived grafts in rats.

Since a key element to the clinical success of autograft bone is the presence of osteogenic cells, we compared the colony-forming and osteogenic capacity of bone marrow cells derived from the femur and ilium. Similar to our observations, previous animal studies in dogs and pigs observed higher CFU-F frequency in the bone marrow derived from the iliac crest than from the femur [44, 45]. Comparisons of marrow-derived mesenchymal cells from human ilia and femurs, however, have revealed conflicting results, with some yielding similar properties [46–51], while others have observed higher concentrations of osteogenic progenitors in marrow from the iliac crest [52, 53] or from the femur [54, 55]. These seemingly contradictory results may largely be due to differences in study design, particularly: the isolation methods used; the specific regions of the femur and hip from which cells are isolated, e.g., proximal vs. distal femur, iliac crest vs. anterior superior iliac spine; and the patient populations under investigation.

One of the key limitations of this study was the high variability observed, particularly in the CT fusion volumes. While it is possible that subtle differences in surgical technique may have influenced this variation, the range in age and weight of the animals used as donors and hosts is a more likely contributing factor, as our previous meta-analysis of rat fusion models showed an association between animal age and/or weight and fusion outcomes [2]. Another potential source of variability, especially in the allograft groups, is the possibility that there were slight within group differences in implanted graft composition; for example, one animal may have received more of the epiphysis portion and another more of the diaphysis portion of the femur. Studies in humans, for example, have shown that the proximal femur contains bone marrow with a higher concentration of osteogenic CFU-F than the distal femur [51]. Although the observed rates of fusion in this study were low compared to several previous rat studies, our allograft hip fusion rate was comparable to the only other study to use freshly isolated iliac allograft, which observed manual palpation fusion rates of ~ 40% in Sprague-Dawley rats [8]. Possible reasons for the lower fusion rates observed in this study include the stringency of our fusion assessment criteria (i.e., only counting solid bilateral fusion) compared to previous studies; the amount of bone graft that we implanted may not have been sufficient to yield higher fusion rates; and rat strain differences in bone healing. In order to optimize the syngeneic allograft rat lumbar fusion model, future studies will control more carefully for these factors, using a tighter age and weight range in donor and host rats, and will examine increasing volumes of bone graft, site-specific bone graft regions (i.e., epiphysis vs. diaphysis), and a wider range of donor bone types.

## Conclusions

Despite limitations, this study demonstrates that employing inbred rat strains enables syngeneic allograft bone to be used successfully in place of autograft bone in lumbar fusion studies, thus reducing donor site morbidity and surgical time in host animals. Furthermore, syngeneic allograft was shown to perform comparably or superiorly to autograft bone in the rat fusion model. More importantly, as the first study to compare freshly isolated hip and femur-derived bone grafts in a spinal fusion model, we demonstrated that while femoral grafts exhibited similar CT fusion mass volumes, iliac grafts displayed both a higher rate of solid radiographic fusion as well as bilateral fusion via manual palpation. In our rat model, this increase in mechanical fusion was associated with superior histological integration with host bone, which was likely connected to the higher observed concentration of osteogenic progenitor cells in hip-derived bone marrow. These results suggest the need for further comparison of femur-derived grafts, such as RIA femoral autograft, and iliac crest grafts in the context of spinal fusion.

## Data Availability

The datasets used and/or analyzed during the current study are available from the corresponding author on reasonable request.

## Abbreviations

CFU-F: Colony-forming unit fibroblast
CT: Computed tomography
DMEM: Dulbecco’s Modified Eagle Medium
PBS: Phosphate-buffered saline
P1: Passage 1
RIA: Reamer–irrigator–aspirator
